# Colocalization analysis reveals shared genetic loci contributing to gout and metabolite levels

**DOI:** 10.3390/gucdd3020006

**Published:** 2025-03-27

**Authors:** Riku Takei, Nicholas A. Sumpter, Megan P. Leask, Tony R. Merriman

**Affiliations:** 1 Division of Clinical Immunology and Rheumatology, University of Alabama at Birmingham, Birmingham, AL, United States; 2 Department of Internal Medicine and Radboud Institute of Molecular Life Science, Radboud University Medical Center, Nijmegen, The Netherlands; 3 Department of Physiology, University of Otago, Dunedin, New Zealand; 4 Department of Microbiology and Immunology, University of Otago, Dunedin, New Zealand

**Keywords:** Gout, metabolite, quantitative trait loci, GWAS, Mendelian randomisation

## Abstract

Genetic association studies in gout have identified genetic variants in or near genes involved in the biosynthesis and transport of urate and in immunological pathways. However, the causal role of the remaining genetic variants, genes, and pathways in gout is not clear. Here, we present the results from a genetic colocalization analysis of gout-associated signals with metabolite quantitative trait loci (mQTL), shedding light on the metabolites that are likely directly affected by genetic variants associated with gout. We identified 141 candidate metabolites with evidence of colocalization with at least one gout-associated genetic signal, of which 29 showed evidence of a causal relationship with gout by Mendelian randomization. Among the 29 metabolites were lysophosphatidylcholines, which may affect the inflammatory response by binding to the TLR-2/4 receptors, providing plausible candidate metabolites for future studies that link metabolites with inflammatory processes in gout.

## 1. Introduction

Gout is a common inflammatory arthritis caused by an innate immune response to monosodium urate (MSU) crystals that primarily deposit in joints [1]. Though hyperuricemia is a prerequisite of gout, an inflammatory response by the NLRP3-inflammasome is required to trigger gouty inflammation [1,2]. Genome-wide association studies (GWAS) in gout [3–8] have revealed many genetic loci, some of which have been implicated in biosynthesis and transport of urate. However, definitive implication of causal pathways of gouty inflammation is not clear (aside from genetic variants linked to innate immune cytokines that have an obvious causal link) [4]. Many genetic studies have linked the genetic signals to other functional information (such as expression quantitative trait loci (eQTL)), but very few have explored causal connections with metabolite levels [9–11].

Metabolomics is a field that involves the measurement of a large number of metabolites within a group of participants to identify metabolite levels that are associated with a phenotype of interest [12,13]. When these cohorts also contain genetic information it allows for the association of genetic variants with metabolite levels [14–16] and provide valuable insights into the biological mechanisms by narrowing the search space for causal metabolites that may be causally related. Recently, there have been several GWAS of metabolomic profile that associated genetic variants with metabolite levels: Chen et al. [14] studied 1,091 blood metabolites and 309 metabolite ratios in 8,299 individuals from the Canadian Longitudinal Study on Aging (CLSA) cohort, Yin et al. [16] studied 1,391 metabolites in the plasma of 6,136 male participants from the Metabolic Syndrome in Men (METSIM) study, and Schlosser et al. [15] studied 1,296 plasma and 1,399 urine metabolites from 5,023 participants from the German Chronic Kidney Disease (GCKD) study. These studies are currently the largest metabolite quantitative trait loci (mQTL) data and provide a wealth of resources to study the effect of genetic variations that influence metabolite concentrations in both plasma and urine, which in turn can be used for insights into the biological mechanism of a phenotype of interest.

In the context of gout, however, only a few metabolomics studies have been done; of note are three studies by Renaudin et al. [9], Lyu et al. [10], and Joshi and McCormick et al. [11]. Renaudin et al. [9] explored the metabolic profile of bone marrow-derived macrophages (BMDMs) and THP-1 cells stimulated with either MSU crystal or calcium pyrophosphate (CPP) crystal and found that the glycolytic pathway, but not oxidative phosphorylation (OXPHOS), has a pivotal role in the IL-1*β*-mediated inflammatory process, which is a key mediator for the inflammatory response in gout. Lyu et al. [10] recruited subjects in various stages of gouty arthritis including no gout, hyperuricemia, acute gout, intermittently symptomatic gout, and chronic tophaceous gout, to identify biomarkers associated with progression of gouty inflammation. They identified four metabolites (kynurenic acid, N1-Methyl-2-pyridone-5-carboxamide, DL-2-Aminoadipic acid, and 5-hydroxyindole acetic acid) as potential biomarkers for progression of gout. More recently, Joshi et al. [11] utilized metabolomics data from the UK Biobank (UKB) cohort to investigate the metabolomic changes that predict incident gout, using Mendelian randomization to identify glycoprotein acetyls (GlycA) as a causal metabolite of incident gout. Lower levels of glutamine were associated with increased risk of gout in both Renaudin et al. [9] and Joshi et al. [11], providing consistent evidence of the role of glutamine in the biological process of gout, possibly through glutaminolysis and trained immunity from the accumulation of fumarate [17]. In fact, the most recent genetic study of gout [4] identified that the proline allele of the p.Leu581Pro missense variant of the glutaminase 2 (GLS2) enzyme, which associated with reduced levels of glutamine, also associates with increased risk of gout, supporting the findings from these metabolomics studies that show the important role of glutamine in gout.

Even though these studies provide valuable insights into the biological processes surrounding gout, only a handful of metabolites have been identified and causally associated with gout and progression of gout, most likely due to several factors including sample size and the onerous number of metabolites being explored (and therefore suffering from limited power owing to multiple testing correction). Furthermore, given that these studies are observational, no conclusions regarding causality are able to be drawn. Since metabolite levels are direct consequences of enzymatic activities, by combining the largest genetic association data of gout [4] and the recent metabolomics studies [14–16] it may be possible to identify metabolites that are also associated with gout-associated genetic variants and consequently identify causal molecular mechanisms of gout. Here we conducted colocalization analyses of genetic loci from the most recent GWAS of gout [4] with the plasma and urine metabolite quantitative trait loci (mQTL) data from CLSA [14], METSIM [16], and GCKD [15] studies in order to identify metabolites that may be affected by genetic loci that also affect gout. Our analysis included pathway enrichment analysis of genes that are associated with both gout and metabolite levels to highlight pathways that are likely involved with the gout-associated metabolites and also include two-sample Mendelian randomization of the metabolites with gout to assess possible causal relationships of the metabolite levels with gout.

## 2. Materials and Methods

### Summary data and meta-analysis

Association summary statistics for 1,400 plasma and plasma metabolite ratios from the CLSA cohort [14], data from 1,391 plasma metabolites from the METSIM study [16], and data from 1,296 plasma and 1,399 urine metabolites from the GCKD study [15] were downloaded from the GWAS Catalog (https://www.ebi.ac.uk/gwas/) and METSIM Metabolomics PheWeb (https://pheweb.org/metsim-metab/). Full European gout and urate GWAS summary statistics from Major et al. [4] were used in all of the analyses. For the purpose of Mendelian randomisation, fixed-effect meta-analysis was carried out using METAL [18] on the 141 metabolites that colocalized with gout genetic signals and that were also present in all three metabolite data sets.

### Colocalization analysis

Given a reigon of interest, genetic colocalisation of two traits tests five hypotheses:

1. Null hypothesis (H0): No association in either traits
2. Hypothesis 1 (H1): Association in first trait only
3. Hypothesis 2 (H2): Association in second trait only
4. Hypothesis 3 (H3): Association in both traits, but the associations do not overlap
5. Hypothesis 4 (H4): Association in both traits and associations overlap (i.e. colocalizes)

Each hypothesis is assigned posterior probability based on how likely the hypothesis describes the data and all posterior probabilities from the hypotheses sum to 1. Therefore, the hypothesis with the highest posterior probability is the most likely scenario that describes the comparison of the two traits. For the purpose of this paper, hypothesis 4 (H4) is of interest as this represent the evidence of colocalization of gout and mQTL association signals and provides evidence for shared genetic etiology. Genetic loci for colocalization analysis were restricted to 276 genetic loci that have prior evidence of association with gout [4]. The regions for colocalization was restricted to 1Mb region cetered around the lead variant (lead variant ±500kb window) and only the variants (based on rsID) present in both the gout GWAS and mQTL data were kept. Colocalization of gout summary statistics with each of the mQTL association summary statistics was carried out using the ‘coloc’ [19] R [20] package for the 276 loci. Loci that contained less than 100 variants within the ±500kb window of colocalization region were excluded from the analysis. A locus was considered to be colocalized if the posterior probability of colocalization (hypothesis 4) was 0.8.

### Mapping of metabolite names

Not all of the metabolite names used in the three mQTL data sets were consistent, so the metabolite names were mapped to those in the Human Metabolome Database [21] (HMDB version 5.0; downloaded from https://hmdb.ca/downloads). The original metabolite name in each of the data set was matched with the metabolite name (or its synonym) as shown in HMDB. If a metabolite name did not match exactly with the HMDB metabolite name, then the name was matched by approximate string matching using the ‘fedmatch’ [22] package in R. In cases where a metabolite name matched with multiple candidate names, the names were checked manually in order to determine the metabolite name that was most likely correct based on chemical structure.

### Pathway enrichment analysis

Protein-coding genes were identified from the gout loci (lead variant ±500kb window) that colocalized with any of the 141 metabolites common to all three mQTL data sets. All protein-coding genes that had their gene region overlapped with the 1Mb region around the lead variant were considered for the pathway analysis. For the identification of genes directly involved with the colocalized metabolite, metabolites from the pathways that involved the colocalized metabolites were identified from the HMDB, after removing metabolites that are “inorganic” (e.g. water) and those present in majority of the metabolic pathways (e.g. ATP is present in 97.8% of pathways, and therefore will be involved in almost all up- and downstream metabolic pathways and add irrelevant genes that uses ATP as a substrate). The list of genes was used for pathway enrichment analysis using DAVID with “Homo sapiens” set as the background [23,24].

### Mendelian randomization

By taking a select variants that are associated with the exposure of interest, Mendelian randomization (MR) tests whether the associated genetic variants (instrumental variable) causes the outcome through the exposure of interest, thus allowing for the testing of causality of the exposure and the outcome. 141 metabolites that colocalized with gout and were present in all three metabolite data sets were considered for MR analysis to determine their causal relationship with gout using the ‘MendelianRandomization’ [25] package in R. Lead variants from each of the 141 meta-analyzed metabolite summary statistics were identified using the –clump option in PLINKv1.90 [26], using the European ancestry samples from the 1000 Genomes Project [27] as the reference for linkage disequilibrium (LD). To ensure that the lead variants identified were LD-independent, pairwise LD was calculated for all of the lead variants using PLINK and 1000 Genomes Project European ancestry data, and variants with *r*^2^ ≥ 0.01 were removed. Any metabolite that had two or less genetically-associated lead variants was not considered for the MR analysis due to technical limitations of the MR methods used. Inverse variance-weighted (IVW) and weighted median (WM) methods were used to test for causality of the metabolite with gout (and vice versa), and the MR-Egger method was used to test for pleiotropy by considering the MR-Egger intercept. The lead variants from gout and serum urate GWAS from the Major et al. [4] were identified using the same approach as above.

## 3. Results

### 3.1. Genetic colocalization of gout and metabolite quantitative trait loci

Colocalization analysis was first carried out for 1,391 plasma metabolites from the METSIM cohort with 276 independent genetic loci from the European gout GWAS from Major et al. [4] (Supplementary table S1). In total, 633 metabolites (45.5% of tested metabolites) colocalized with at least one gout locus implicating 135 (48.9%) of the 276 tested loci, highlighting the extent to which gout genetic loci associate with the human metabolome (Table 1). The proportion of metabolites (45.5%) and genetic loci (48.9%) are comparable to the proportion observed in other studies that colocalized mQTL with disease traits [28,29]. On average, a metabolite colocalized with 1.56 gout loci in plasma, and there were no more than six colocalized loci for any one plasma metabolite. Conversely, the average number of metabolites that colocalized with a particular gout locus was 7.12, with the largest number of metabolites (n = 178) colocalizing at the *GCKR* locus (chr2:26.91MB-28.71MB). These results suggest that metabolites are genetically controlled by a small number of gout-associated loci while a gout genetic locus is able to influence a variety of metabolites.

We repeated the colocalization analysis in metabolite data sets from two independent cohorts: CLSA and GCKD (Table 1 and Supplementary tables S2–S4). Similar to what was observed with the METSIM cohort, 137 (49.6%) gout loci colocalized with 719 (51.4%) metabolites from the CLSA cohort (Table 1; 7.96 metabolites per gout locus). In the GCKD cohort, the number of loci colocalized with plasma and urine metabolites were comparable to the number observed in METSIM (136 (49.3%) and 138 (50.0%) loci), but with less metabolites compared to the METSIM and CLSA cohorts (482 (37.2%) plasma and 430 (30.7%) urine metabolites; 4.74 and 4.17 metabolites per locus, respectively). Furthermore, the average number of gout loci that colocalized with a particular metabolite was comparable to that observed in the METSIM cohort (1.55 in the CLSA cohort, 1.36 and 1.34 loci with plasma and urine metabolites in the GCKD cohort, respectively). Compared to the METSIM data set, the average number of metabolites that colocalized with a gout locus was slightly larger in the CLSA cohort (7.96 metabolites) and lower in the GCKD cohort (4.74 for plasma and 4.17 for urine metabolites), though this may be due to the lower total number of metabolites that colocalized with the gout loci in the GCKD data set. However, the overall trend where there are more colocalized metabolites per locus compared to the number of gout loci that colocalize with a metabolite was consistent across cohorts.

**Figure 1.**
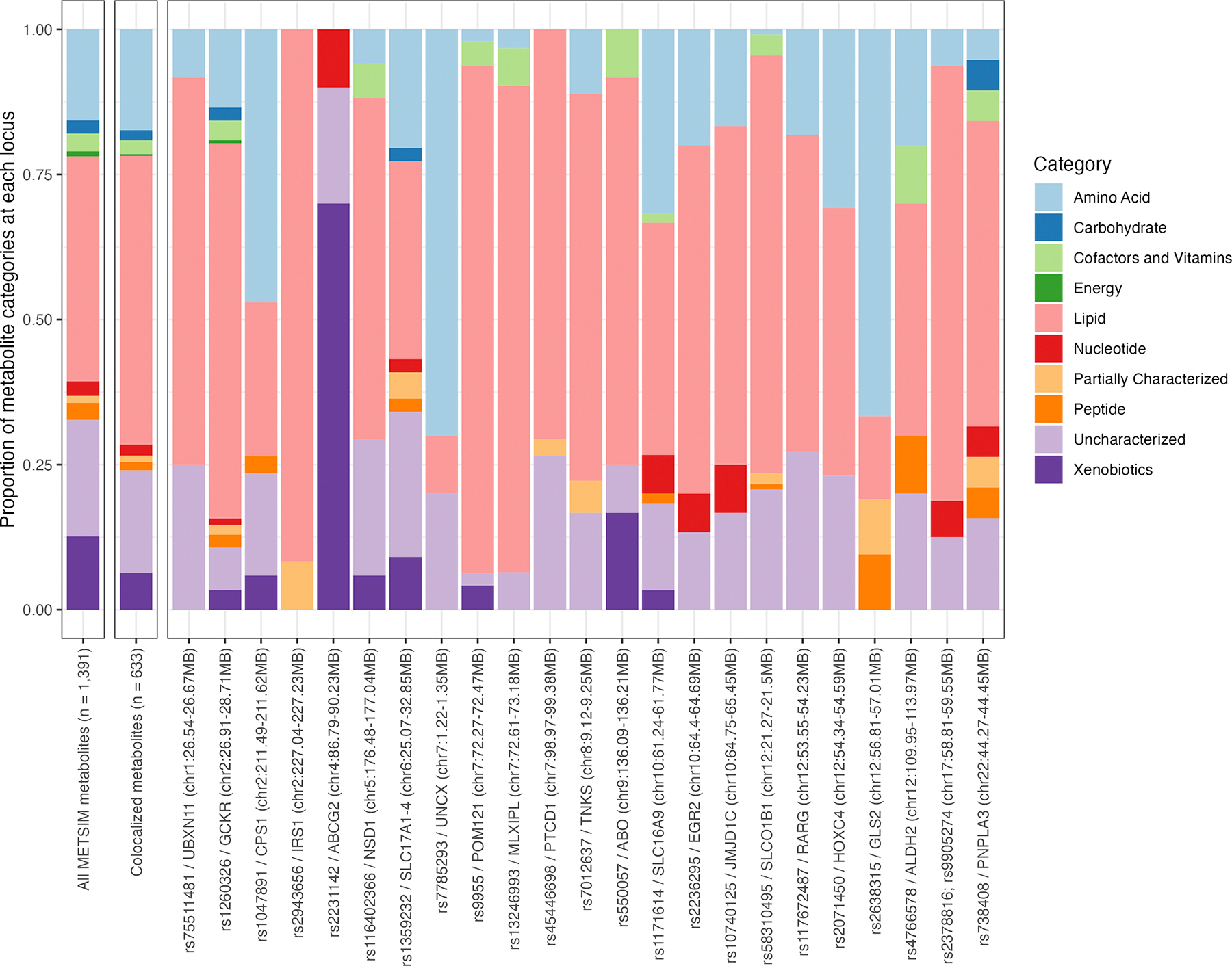
Proportion of broad categories for all 1,391 METSIM metabolites (left panel), 633 colocalized metabolites (middle panel), and 23 gout loci with at least ten colocalized metabolites (right panel).

Next, we investigated the types of metabolites that colocalized at each of the gout loci in the METSIM cohort using the ten category descriptions (amino acids, carbohydrate, cofactors and vitamins, energy, lipid, nucleotide, partially characterized, peptide, uncharacterized, and xenobiotics; Supplementary table S5). Among the 633 metabolites that colocalized with a gout locus, lipid metabolites were the most common (proportion of metabolites that were lipids = 0.498), followed by uncharacterized metabolites (0.177), and amino acids (0.174). These proportions were similar to the overall proportions observed in all METSIM metabolites (n = 1,391 metabolites; Figure 1 and Supplementary table S5). The proportions from the colocalized metabolites were similar in loci that had >10 colocalized metabolites (23 loci in total), except at the *ABCG2* locus (rs2231142, chr4:86.79MB-90.23MB; Figure 1). At the *ABCG2* locus, 70% (7/10) of the metabolites that colocalized were xenobiotics, likely reflecting the role of ABCG2 as a universal transporter for a variety of drugs, metabolites, and molecules [30], including urate (one of the colocalized metabolites at this locus and causal of gout). This result was not able to be compared with the CLSA and GCKD cohorts, as the category information was not available in these metabolite datasets.

### 3.2. Pathway enrichment analysis of genes at loci that colocalized with metabolite levels

**Figure 2.**
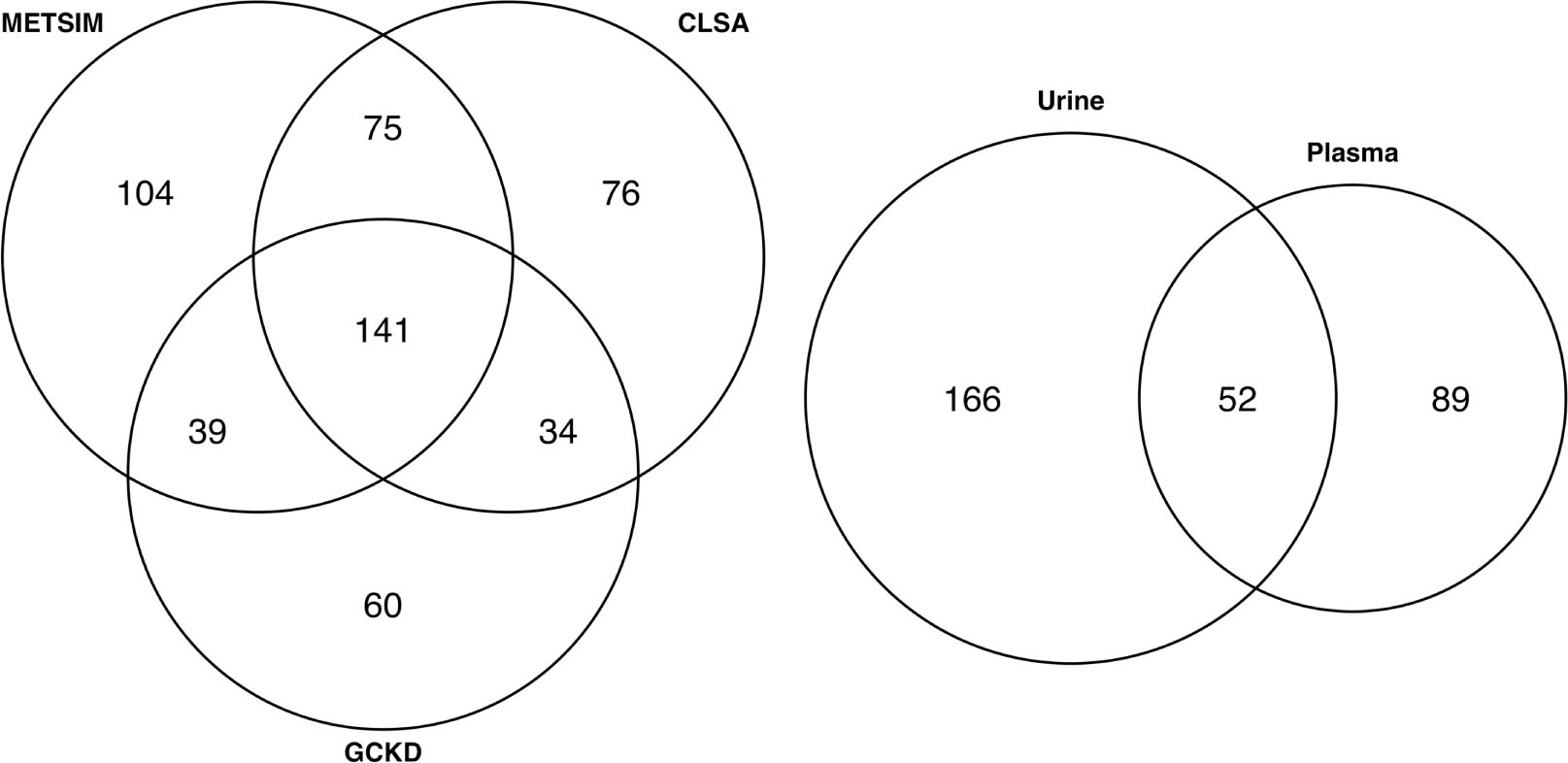
Venn diagram showing the overlap of the colocalized plasma metabolites in METSIM, CLSA, and GCKD cohorts (left), and the overlap of 141 common metabolites with colocolised GCKD urine metabolites (right).

To identify plasma metabolites that were colocalized in the three data sets (METSIM, CLSA, and GCKD), the metabolite names from each data set were first mapped to standard metabolite names from the Human Metabolome Database (HMDB; Methods). In total, 359 (359/633 = 56.7%), 326 (326/719 = 45.3%), and 274 (274/482 = 56.8%) plasma metabolites from METSIM, CLSA, and GCKD cohorts matched with an HMDB metabolite name, respectively, of which 141 (141/529 = 26.7%) metabolites were common in all three data sets (Figure 2 and Supplementary table S6). Of the 141 common plasma metabolites, 89 (63.1%) did not colocalize in urine (out of 218 urine metabolites that had a HMDB metabolite name; Figure 2 and Supplementary tables S7 and S8).

To determine the biological pathways represented by the colocalized metabolites, pathway enrichment analysis was carried out using the genes present in the loci that colocalized with 141 plasma metabolites that were common among all three data sets. Three sets of protein-coding genes (1,489, 1,145, and 908 genes) were identified from the loci that colocalized with all 141 plasma, 89 plasma-specific, or 52 metabolites present in both plasma and urine, respectively (Methods). Fifteen, eight, and nine pathways were significantly enriched (FDR-adjusted P ≤ 0.05) with the three gene sets (Supplementary tables S9–S11), respectively. Notable pathways identified from plasma-specific genes included glycolysis/gluconeogenesis, pyruvate metabolism, ethanol oxidation, and retinol metabolic process, indicating the genes present in the loci that colocalized specifically with the plasma metabolites may be derived from metabolic processes for energy generation. In contrast, pathways that were enriched by the genes present in the loci that colocalized with both plasma and urine mainly included transportation, rather than metabolism, of molecules and compounds, such as urate metabolism and transport, transmembrane and organic anion transport, and bile transport and secretion. Many of the plasma-specific and plasma and urine pathways were present in the full list of genes (set 1), though there were some unique pathways that did not appear in the other two sets including the PI3K-AKT signaling pathway, and the insulin resistance and signaling pathway.

**Figure 3.**
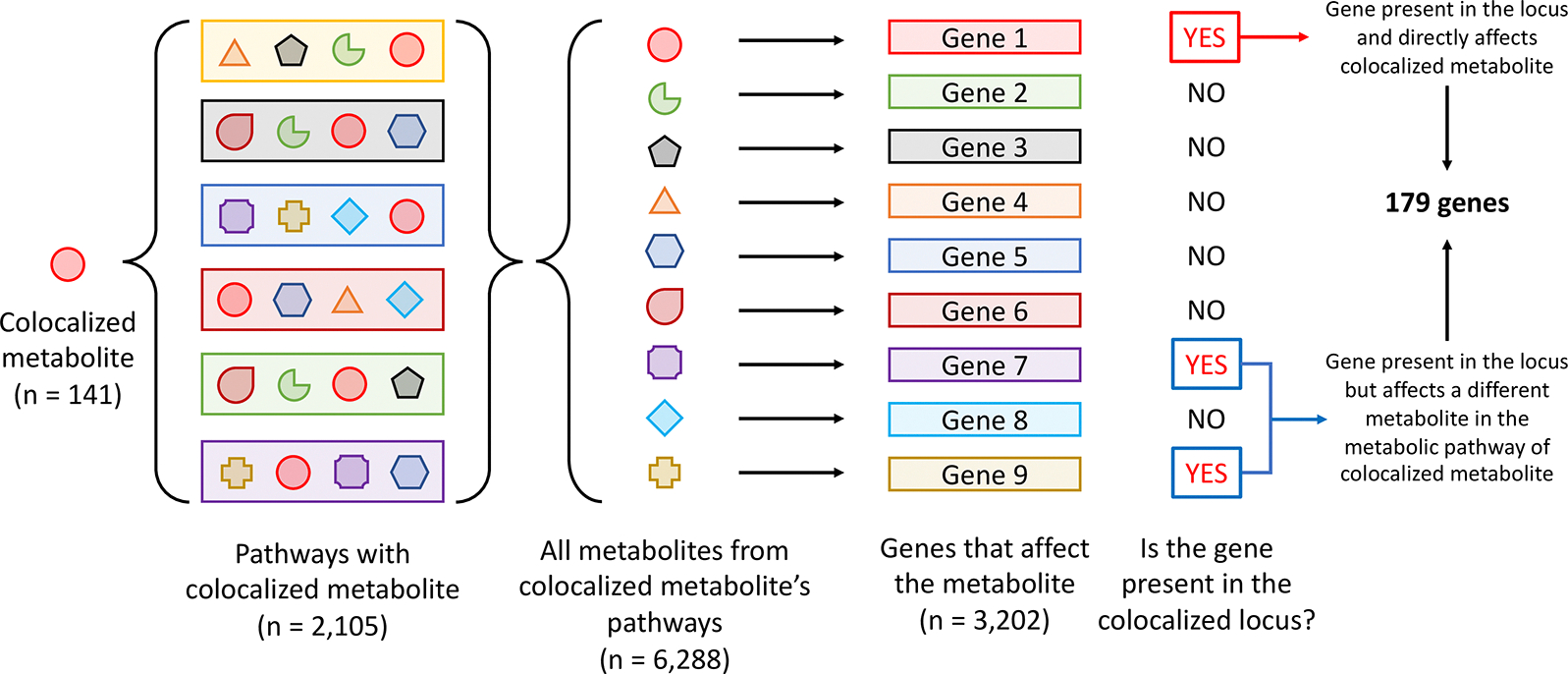
Schematic of the identification of metabolites directly affected by genes present in the gout loci that colocalized with metabolite levels.

Given that not all of the genes will be directly involved with the biological pathways that affect the metabolite level, we focused on identifying genes directly involved with generation of the 141 colocalized plasma metabolites (Figure 3). We first identified 2,105 pathways that included at least one of the 141 plasma metabolites based on the pathway information in HMDB and extracted 6,288 metabolites from these pathways to ensure that any upstream or downstream metabolites, and therefore the genes that may affect the level of the 141 plasma metabolites, were included. Based on the information available in the Human Metabolome Database (HMDB), 3,202 genes encoding enzymes and proteins that directly affected the 6,288 metabolites were extracted and matched back to the loci that colocalized with the 141 plasma metabolites, of which 179 genes were located within the colocalized loci. This list of 179 genes is the most relevant set of genes that may affect the 141 plasma metabolite levels directly or indirectly, as the protein products of these genes affect either the colocalized metabolite itself, or affect the metabolite that is up- or downstream to the metabolite of interest in a pathway (Supplementary table S12). Of the 179 genes, however, only 17 at 9 gout loci encoded proteins that directly metabolizes or generates the colocalized metabolite; for example, the *GLS2* (encoding glutaminase 2) was identified at the rs58310495 locus (chr12:21.27MB-21.5MB region) which colocalized with L-glutamine (Supplementary table S13). Pathway enrichment analysis of the 179 genes identified 71 enriched pathways (FDR-adjusted P ≤ 0.05), of which 63 were not identified in the previous analyses, though some were closely related, such as fatty acid metabolism, metabolism of carbohydrates, and transport of organic anions (Supplementary table S14). Among the 63 significant pathways were estrogen metabolic process, androgen biosynthetic process, steroid biosynthesis, mitochondrial fatty acid beta-oxidation, a variety of amino acid metabolic processes (including, but not limited to, glutamine, arginine, proline, valine, leucine, and isoleucine), metabolism of water-soluble vitamins and cofactors.

### 3.3. Mendelian randomization of colocalized metabolites with gout and urate

Lastly, two-sample Mendelian randomization (MR) analysis was carried out to evaluate causality of the plasma metabolites with gout. In order to focus the search for metabolites that are most likely causal for gout, we focused on the 141 plasma metabolites that had mQTL colocalized with gout genetic association signals that were common amongst the three plasma metabolite data sets. The summary statistics of all 141 plasma metabolites of the three data sets were meta-analyzed and genetically-independent lead variants genetically associated with the level of each metabolite were identified (Methods). Of the 141 plasma metabolites, 126 metabolites had at least three independent lead variants for the MR analysis (Methods). 29 metabolites (including urate) showed evidence of causality with gout (Bonferroni-corrected P ≤ 3.97×10^−4^) in either the inverse variance-weighted (IVW) or weighted median (WM) methods, of which four metabolites (3,5-dichloro-2,6-dihydroxybenzoic acid, mannose, myo-inositol, and perfluorooctanoate) showed evidence of pleiotropy (MR-Egger intercept P of 0, 2.31 × 10^−14^, 3.05 × 10^−11^, and 0, respectively; Supplementary table S15). Urate was not pleiotropic with gout (MR-Egger intercept P = 0.32), confirming the causal mechanism of gout through urate and not via other confounding factor. Among the 29 metabolites were lysophosphatidylcholines (LPCs; such as 1-oleoyl-GPC, 1-palmitoleoyl-GPC, and 1-palmitoyl-2-palmitoleoyl-GPC), acylcarnitines (such as (R)-3-hydroxybutyrylcarnitine and 3-hydroxyhexanoylcarnitine), amino acid derivatives (such as gamma-glutamylglutamine, N-acetylalanine, and N-acetylglycine), and sugar molecules (such as mannose and myo-inositol).

The effect estimates of two of the three acylcarnitines ((R)-3-hydroxybutyrylcarnitine and 3-hydroxyhexanoylcarnitine) were in a positive direction, indicating that the increased level of these metabolites increase the risk of gout. In contrast, all three lysophosphatidylcholines (1-oleoyl-GPC, 1-palmitoleoyl-GPC, and 1-palmitoyl-2-palmitoleoyl-GPC) showed negative effect estimates, suggesting a protective role of LPCs in gout. To evaluate reverse causality, that is gout having a causal effect on the metabolite level, we reversed the MR analysis to test the causality of gout on the metabolite levels and found urate to be the only metabolite that had evidence of causal relationship with gout (P_*IVW*_ =3.38×10^−4^ and P_*WM*_ =7.77×10^−3^; Supplementary table S16). However, gout also had evidence of pleiotropy with urate (MR-Egger intercept P = 5.67 × 10^−4^), reflecting the fact that the gout variants affect both urate levels and gout, making it difficult to determine the direction of causality. Since urate is a known causal risk factor of gout, we also tested the causality of these 28 metabolites (29 metabolites sans urate) with Major et al. [4] urate GWAS and found 24 of 28 metabolites with evidence of causality (Bonferroni-corrected P ≤1.79×10^−3^; Supplementary table S17), with gamma-glutamylthreonine, glycocholenate-sulfate, N-acetylalanine, and octadecanedioate showing no evidence of causality with urate. There were six metabolites that showed evidence of pleiotropy (1-oleoyl-GPC, eicosanedioate, homocitrulline, mannose, myo-inositol, and perfluorooctanoate; Supplementary table S17). Testing for reverse causality, there was no evidence of urate having causal effect on the levels of the 28 metabolites (Supplementary table S18), thus 18 metabolites (24 urate-associating metabolites – 6 pleiotropic metabolites) may be causal of gout via their effect on urate.

## 4. Discussion

**Figure 4.**
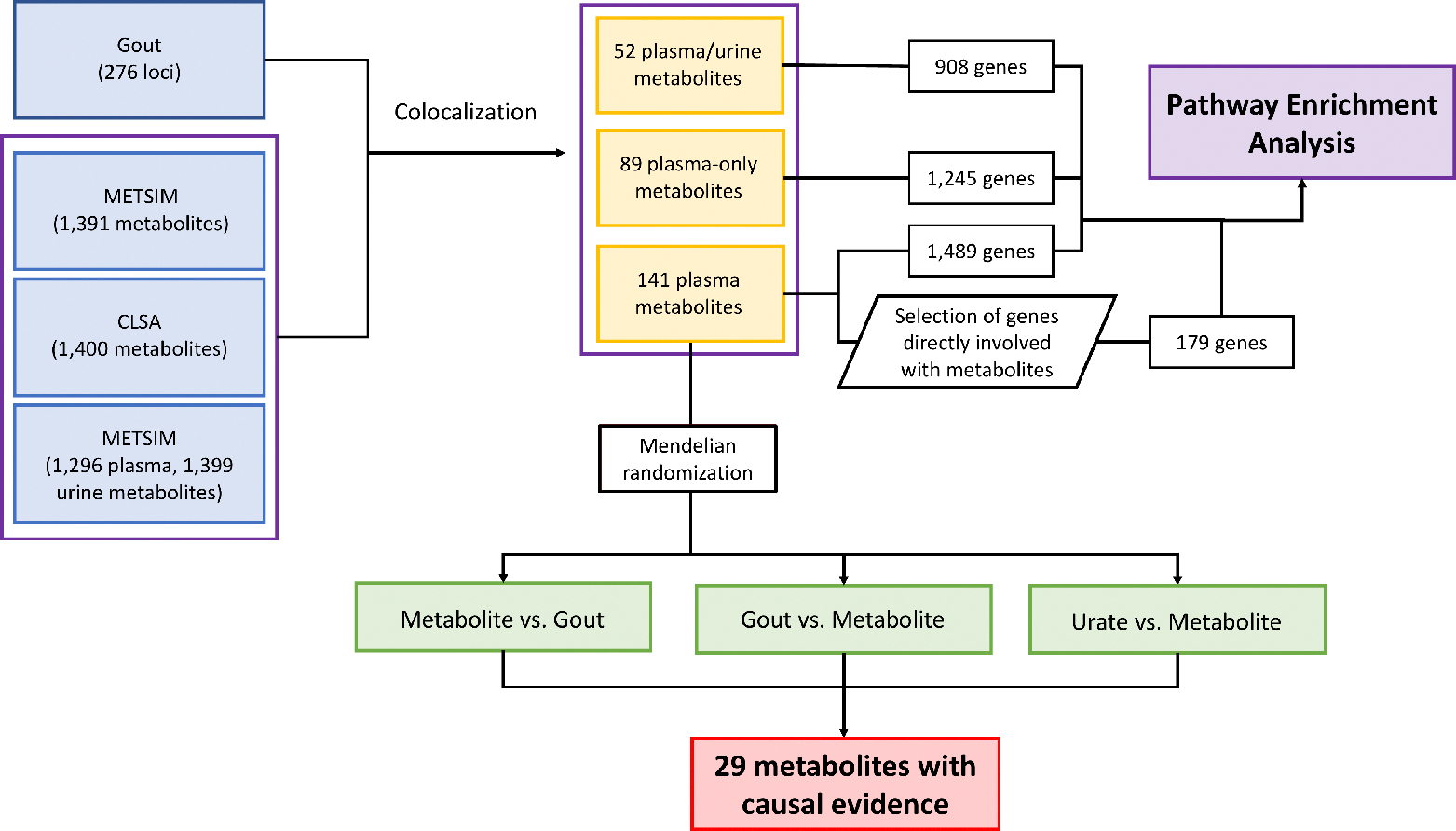
Summary of the analyses carried out in this paper including colocalization analysis, pathway enrichment analysis, and Mendelian randomization analysis.

In this study, we explored the association of metabolite levels with gout by first considering the genetic loci that were identified from the most recent and largest gout GWAS to date, thus narrowing down the search space for candidate metabolites that may be important in gout, and narrowing to metabolites that have some genetic implication in gout. The summary of the analyses is shown in Figure 4. We observed an average of 7.12 metabolites genetically colocalizing with each gout locus and an average of 1.56 gout loci colocalizing with each metabolite, highlighting the fact that a gout genetic locus likely affects several metabolites within the same metabolic pathway, but each metabolite is directly affected by one, or at most a few, gout genetic loci (Table 1). We then identified 179 genes present in the colocalized loci that directly affected the breakdown or production of metabolites within the pathways that involved the 141 plasma metabolites that colocalized in all three mQTL data sets (Supplementary table S12). Surprisingly, among the 179 genes, there were only 17 genes at 9 gout loci that directly metabolized or generated the colocalized metabolites. This suggests that genetic loci identified from GWAS rarely include genes that encode proteins that directly affect the metabolite levels. The 179 genes that were curated from this approach yielded an additional 63 pathways that were not identified by using the list of all of the genes present in any of the colocalized gout loci (Supplementary table S14). For example, estrogen, androgen, and steroid biosynthesis pathways were three of the pathways identified from the analysis. Gout is a disease manifested mainly in men [1,31] and the fact that we identified several pathways that are related to sex hormones and steroid biosynthesis supports these pathways as an underlying biological mechanism that puts men at a higher risk for gout.

Other pathways of note are the amino acid metabolic processes, in particular the glutamine metabolic process. Glutamine has been identified as associated with gout in published metabolomics studies [9,11] and has been reported to be one of the key metabolites in establishing trained immunity via the tricarboxylic acid (TCA) cycle. Trained immunity is a phenomenon where innate immune cells (such as natural killer (NK) cells and monocytes) gain an increased and non-specific immune response by epigenomic remodelling against subsequent infections after being exposed to the initial pathogen [17,32], and many of the substrates, for example fumarate, succinate, and citrate, promote (e.g. citrate converted to acetyl-CoA thus providing the acetyl group for acetylation [33]) or inhibit (e.g. fumarate directly inhibits KDM5 family of histone demethylases [34,35]) the epigenetic reprogramming that is essential for training. Training by soluble urate has also been experimentally implicated in enhanced monocyte response to MSU crystal [36]. This makes glutamine a strong candidate metabolite involved in the inflammatory process of gout. In fact, rs2638315 (chr12:56.81MB-57.01MB) colocalized with glutamine and is in complete LD with the missense variant rs2657879 for *GLS2* [4] (encodes glutaminase 2) which directly metabolizes glutamine (Supplementary table S13), providing an immediate candidate gene that can be tested for functional validation. *CAD* (encoding carbamoyl-phosphate synthetase 2) at the rs1260326 (chr2:26.91MB-28.71MB) locus colocalized and directly involves glutamine, and is involved in the first few rate-limiting steps of de novo pyrimidine biosynthesis [37]. However, it is not clear whether the gout risk is mediated through changes in glutamine levels and pyrimidine biosynthesis pathway by *CAD* or by another more well-established gout gene, *GCKR*, in the same locus, or both.

Mendelian randomization analysis of the 141 plasma metabolites was carried out to establish causal relationship of the metabolites with gout and to identify directionality of association, identifying 29 metabolites with causal evidence for gout (Supplementary table S15 and S16) including lysophosphatidylcholines (LPCs), acylcarnitines, amino acid derivatives, and sugar molecules. LPCs are phospholipids that can directly bind to toll-like receptors (TLR) 4 and TLR-2/1 [38] and, consistent with our MR results, lower plasma level of LPCs have been observed in patients with active rheumatoid arthritis [39,40] and have also been associated with other diseases (such as diabetes, schizophrenia, polycystic ovary syndrome, Alzheimer disease, pulmonary arterial hypertension, aging, asthma, and liver cirrhosis) [41]. However, proposing any inflammatory property of LPCs in gout is complicated as they can either activate or inhibit the TLR-mediated signaling pathway depending on the absence or presence of classical TLR ligands, respectively [38,41]. TLR-2 and TLR-4 receptors are central in the inflammatory response to MSU crystal where, upon stimulation by an external signal, the receptors induce expression of the proteins involved in formation of the NLRP3 inflammasome, thus mediating the cleavage of pro-IL-1*β* to pro-inflammatory cytokine IL-1*β* [42]. There have been no functional experiments conducted for LPCs in the context of gout and establishing their biological role in gout would be of great interest.

Acylcarnitines are a group of molecules formed from carnitine and acyl-coenzyme A (acyl-CoA) and their role is to transport fatty acids from the mitochondrion to the cytoplasm for energy production via *β*-oxidation. In fact, lipid metabolic processes and *β*-oxidation (a process where acylcarnitine is sequentially cleaved to generate acetyl-CoA, which is then fed into the TCA cycle for energy production) were identified in the pathway enrichment analysis, supporting the possible role of acylcarnitines in the pathogenesis of gout. A previous study by Kolz et al. [43] associated DL-carnitine and propionyl-L-carnitine with serum urate via a variant in the *SLC16A9* locus (carnitine efflux transporter), where the variant associated with lower levels of DL-carnitine and propionyl-L-carnitine, and higher serum urate. The TCA cycle has a pivotal role in the establishment of innate immunity [17, 33,34] through epigenomic remodelling, and acylcarnitines, via generation of acetyl-CoA, could be a plausible causal metabolite of gout through trained immunity. Innate immune cells are trained by epigenetic modifications such as mono- and tri-methylation of lysine 4 of histone 3 (H3K4me1 and H3K4me3) and acetylation of lysine 27 at histone 3 (H3K27ac) at cytokine genes such as *TNFA*, *IL6*, and *TLR4* for rapid and non-specific response to subsequent infections [33,44]. In fact, pathway analysis of genes genetically-associated with gout [4] revealed chromatin modifications as one of the significantly enriched pathways and many histone methyltransferase genes were included in the analysis, suggesting an emerging role of histone modifications in gout most likely via trained immunity. Other mechanisms such as glycolysis [34], lipid biosynthesis [45], and the pentose phosphate pathway [17,34] have been implicated in trained immunity, many of which are closely linked to the TCA cycle metabolites. Furthermore, MSU crystal has been demonstrated to act as a danger-associated molecular pattern (DAMP) and elicits an innate immune response and soluble urate can induce epigenetic reprogramming of innate immune cells and contribute to trained immunity [46,47]. Both urate and carnitine derivatives have been causally associated with gout in this study through Mendelian randomization, indicating the possibility of carnitines and urate contributing to trained immunity through the TCA cycle and/or altered levels of serum urate.

There are several limitations to this study. First, the inconsistent names of the metabolites in different metabolomics studies have made it difficult to consolidate specific metabolites in all data sets. Even though we used HMDB to optimize consistent naming across the data sets, not all metabolites were able to be mapped to the names in HMDB. In fact, only about half of the metabolite names in the studies were able to be assigned a consistent name based on HMDB, thus almost half of the metabolites were not assessed for a possible role in gout. Other than to encourage future metabolomics studies to use a consistent metabolite naming scheme, this will be a limitation for any study that uses multiple publicly available metabolomics studies. A second limitation is the underrepresentation of transporters cataloged in HMDB. In our study, we used the protein information present in HMDB to link the genes (and hence gene product) to the metabolite(s) in order to generate a list of genes that directly affected the 141 plasma metabolites for the pathway enrichment analysis. Since many of the genes that have the strongest association signals in gout are transporters, the consequence of lower representation of transporters in HMDB was large. However, even allowing for the lack of transporter genes, we were able to identify many relevant pathways that involved metabolites that we found to have a causal link to gout from the MR analysis. A third limitation is the difficulty of determining whether the causal metabolites are acting solely through urate or through both urate and the inflammatory response to MSU crystal. Mendelian randomization of metabolite levels on urate was carried out to address this issue (Supplementary table S17 and S18); however, it is difficult to determine with certainty whether the causal mechanism of the metabolites on gout is truly through urate, due to the fact that the the underlying genetics of urate and gout has considerable overlap and it is very difficult to distinguish the loci that specifically affect urate from those that specifically affect the risk of gout. Regardless of whether urate mediates the effect of the metabolite levels on gout risk, the 29 metabolites warrant further functional investigation to determine their roles in the mechanism of gout. Lastly, due to the nature of metabolomics studies it is very difficult to gather a large cohort and the sample sizes of the metabolomics studies were relatively small compared to traditional GWAS. Since there were less samples, the power to detect association signals was lower and posed a challenge with lead variant selection for an instrumental variable for association with gout. We ameliorated this by meta-analyzing the three data sets in order to increase the likelihood of identifying more association signals for the MR analysis. In the future, we expect to find more metabolites that associate with gout as larger metabolomics studies are performed.

## 5. Conclusions

Metabolomics is a rapidly expanding field in genetics and the wealth of information provided by these studies is extremely valuable. By utilizing the data from metabolomics study, we identified 141 plasma metabolites that genetically colocalized with gout and 29 metabolites that have evidence of a causal relationship with gout, of which acylcarnitines and lysophosphatidylcholines represent strong candidates for future functional studies. These metabolites, especially those that showed causal relationship with gout, have potential as biomarkers.

## Supplementary Material

Supplementary Tables

## Figures and Tables

**Table 1.** Summary of the number of metabolites and gout genetic loci colocalized with one another

Study	Total number of metabolites	Number of unique metabolites colocalized with gout loci	Number of gout loci colocalized with metabolites	Average number of metabolites that colocalized with gout locus	Average number of loci that colocalized with a metabolite

METSIM	1,391	633	135	7.12	1.56
CLSA	1,400	719	137	7.96	1.55
GCKD (plasma)	1,296	482	136	4.74	1.36
GCKD (urine)	1,399	430	138	4.17	1.34

* Note that the average number of metabolites per gout locus includes metabolites colocalized to multiple loci.

## Data Availability

Association summary statistics for 1,400 plasma and plasma metabolite ratios from the CLSA cohort (GCST90199621–90201020) [14] and data from 1,296 plasma and 1,399 urine metabolites from the GCKD study (GCST90264176–GCST90266872) [15] were downloaded from the GWAS Catalog (https://www.ebi.ac.uk/gwas/). Data from 1,391 plasma metabolites from the METSIM study [16] was downloaded from the METSIM Metabolomics PheWeb (https://pheweb.org/metsim-metab/). Data from the Human Metabolome Database [21] was downloaded from https://hmdb.ca/downloads (HMDB version 5.0). All code used in this paper is available at https://github.com/rikutakei/metqtl_code.

## Abbreviations

The following abbreviations are used in this manuscript:

CLSA: Canadian Longitudinal Study on Aging
FDR: False-Discovery Rate
GCKD: German Chronic Kidney Disease
GWAS: Genome-Wide Association Study
HMDB: The Human Metabolome Database
LD: Linkage Disequilibrium
METSIM: Metabolic Syndrome in Men
MSU: Monosodium Urate
QTL: Quantitative Trait Loci
